# MapReduce-Based Parallel Genetic Algorithm for CpG-Site Selection in Age Prediction

**DOI:** 10.3390/genes10120969

**Published:** 2019-11-25

**Authors:** Zahra Momeni, Mohammad Saniee Abadeh

**Affiliations:** 1 Faculty of Electrical and Computer Engineering, Tarbiat Modares University, Tehran P.O. Box 14115-143, Iran; z_momeni@modares.ac.ir; 2 Institute for Research in Fundamental Sciences (IPM), School of Computer Science, Tehran P.O. Box 14115-143, Iran

**Keywords:** age prediction, MapReduce, parallel genetic algorithm, CpG-site selection, GBR Model

## Abstract

Genomic biomarkers such as DNA methylation (DNAm) are employed for age prediction. In recent years, several studies have suggested the association between changes in DNAm and its effect on human age. The high dimensional nature of this type of data significantly increases the execution time of modeling algorithms. To mitigate this problem, we propose a two-stage parallel algorithm for selection of age related CpG-sites. The algorithm first attempts to cluster the data into similar age ranges. In the next stage, a parallel genetic algorithm (PGA), based on the MapReduce paradigm (MR-based PGA), is used for selecting age-related features of each individual age range. In the proposed method, the execution of the algorithm for each age range (data parallel), the evaluation of chromosomes (task parallel) and the calculation of the fitness function (data parallel) are performed using a novel parallel framework. In this paper, we consider 16 different healthy DNAm datasets that are related to the human blood tissue and that contain the relevant age information. These datasets are combined into a single unioned set, which is in turn randomly divided into two sets of train and test data with a ratio of 7:3, respectively. We build a Gradient Boosting Regressor (GBR) model on the selected CpG-sites from the train set. To evaluate the model accuracy, we compared our results with state-of-the-art approaches that used these datasets, and observed that our method performs better on the unseen test dataset with a Mean Absolute Deviation (MAD) of 3.62 years, and a correlation (R^2^) of 95.96% between age and DNAm. In the train data, the MAD and R^2^ are 1.27 years and 99.27%, respectively. Finally, we evaluate our method in terms of the effect of parallelization in computation time. The algorithm without parallelization requires 4123 min to complete, whereas the parallelized execution on 3 computing machines having 32 processing cores each, only takes a total of 58 min. This shows that our proposed algorithm is both efficient and scalable.

## 1. Introduction

Aging is a natural and undeniable process in the life of living organisms. This process is affected by various factors such as inheritance, environment, lifestyle and disease [1]. The aging process alters the telomeres, gene expression and cellular structures in living organisms. By evaluating biomarkers, one can find out about the biological changes that occur in the body [2]. Several biomarkers can be used for age predicton. One of the human age-related biomarkers is DNA methylation (DNAm), which is biologically and chemically more stable than biomarkers such as RNA messenger (mRNA) and proteins [2], and among these biomarkers, it is also more correlated with age [3].

DNAm, which often occurs in a CpG sequence, is an epigenetic sign and plays an important role in regulating cells to establish and maintain cellular identity. Various studies have shown that DNAm changes with age [4,5,6,7,8]. This change in DNAm can be disclosed at specific CpG-sites in all individuals, although individual differences may affect the “speed” of this change [6].

Various models for age prediction have been applied to DNAm data such as regression models including Linear regression [9,10], Multivariate linear regression [2,7], Multiple linear regression [3,11], or machine learning algorithms such as Support Vector Regression [1], Random Forest Regression [6], Artificial Neural Networks [12,13], and Gradient Boosting Regression [4,14]. In a vast majority of these studies, the mean absolute deviation (MAD) and the correlation between the actual value of age and DNAm (R^2^) were reported to describe the model’s performance. Furthermore, most of these studies only used a simple statistical feature selection algorithm such as Pearson’s correlation to select age-related CpG-sites [1,4,14]. In general, traditional feature selection algorithms are divided into three categories: filter, wrapper and embedded methods. Filter methods often use statistical functions, and therefore they are among the most rapid feature selection methods. Since these methods do not use a model to evaluate the selected features, they are not accurate enough, and additionally may select redundant features as they do not consider the interactions between features. As a consequence, many informative features may be ignored. For example, Naue et al. [6] used the Mutual Information and Li et al. [4] used the Pearson’s correlation to select a subset of features. In wrapper methods, in contrast to filter methods, a model is used to evaluate a subset of features, so as the data volume increases, the model accuracy and algorithm time complexity are both increase. For instance, Vidaki et al. [12] used stepwise regression method to select 23 CpG-sites out of the 45 CpG-sites. The embedded methods combine the benefits of the two previous methods and select the subset of features during the modeling process with less computation time than wrapper methods. For example, Weidner et al. [7] used an embedded methode, called Recursive Feature Elimination, in order to select five features to evaluate the multivariate linear regression model.

Nonetheless, a proper feature selection method should be used to select a subset of optimal features that are both informative alone and interact well with other selected features. Wrapper methods are a good choice regardless of their timing constraints. However, as the feature search space gets bigger, the runtime also increases by a wide margin. This is a serious problem in genomic data, where the number of features ranges from several tens to hundreds of thousands. There are several solutions for improving execution time of the complex wrapper methods for analyzing genomic data. In a series of studies, wrapper methods are used in combination with fast filter methodes [15,16,17,18]. In this approach, first, the dimensionality of the feature space is reduced from several thousand to several tens of features by taking advantage of a filter feature selection algorithm, and then a wrapper feature selection method is used to choose features from the remaining ones. However, in this way the previous problem still exists since many important features may be removed when reducing the data size from thousands to tens of features. Another set of studies used parallel computing to overcome the challenge of high execution time of feature selection methods. For example, Nieto et al. used the PSO algorithm in a parallel manner to select cancer-related genes in microarray data [19]. In a similar work, Keco et al. used a type of parallel genetic algorithm to select features from microarray data for cancer classification task [20], and Brahim et al. split the data into several partitions and executed feature selection algorithm on each partition independently. Finally, they combined the selected features from each partition and created their model [21]. Islam et al. proposed a scalable parallel gene selection method using the Map Reduce programming model. They generated a predefined number of potential gene subsets of equal sizes, then calculated the classification accuracy of each subset using the KNN algorithm in parallel. Finally, they ranked features based on their existence in best gene subsets and select genes with highest rankings [22].

The goal of this study is to select the most important features in human age prediction using a rapid parallel framework. To achieve this goal, we first divide the training samples into three age groups based on similarity of their age change pattern to get better results. Second, we use the wrapper based genetic algorithm as feature selection method to find a set of features that optimize MAD of regression model for each age group. Third, we take advantage of parallel computing approaches in three parts of our proposed method and change some standard operators of genetic algorithm to lower the execution time. Finally, we evaluate our proposed method using 16 publicly available DNAm datasets, and obtain a MAD of 3.62 for the test data. We observe that the execution time of our proposed method is around 71.08 times lower compared to the sequential mode.

## 2. Materials and Methods

### 2.1. Data Collection and Preproccesing

The datasets used in this paper are collected from the National Center for Biotechnology Information (NCBI) Gene Expression Omnibus (GEO) (https://www.ncbi.nlm.nih.gov/geo/query/acc.cgi). This site is a source for a variety of public genomic data. These datasets were retrieved from two platforms: (1) HumanMethylation27 BeadChip (2) HumanMethylation450 BeadChip. In this paper, we use 16 healthy DNAm datasets from blood tissue, all of which are in matrix format. In these datasets, columns represent CpG-sites and rows represent healthy samples. Table 1 details each dataset.

The number of samples in these datasets is less than 500, while the number of features varies between 250,000 and 450,000. We merge the datasets to obtain enough samples for learning the ages. This leads us to a final set that covers the age range of 0–103 years. The data preprocessing is done by first removing the samples and columns that are completely null. Then, the columns containing a missing value are imputed with the average strategy. Subsequently, the data is normalized, and the outliers are detected and cancelled out. This leaves us with 2079 samples in our pre-processed dataset. We randomly pick two-thirds of the pre-processed dataset as the train set, and use the remaining third as the unseen test data. To validate the model, we perform three-fold cross-validation. The reason that we did not choose more folds for cross-validation is that our dataset only contains a small number of samples per age range and it is possible that some age ranges get omitted in validation.

### 2.2. Genetic Algorithm

**Figure 1:**
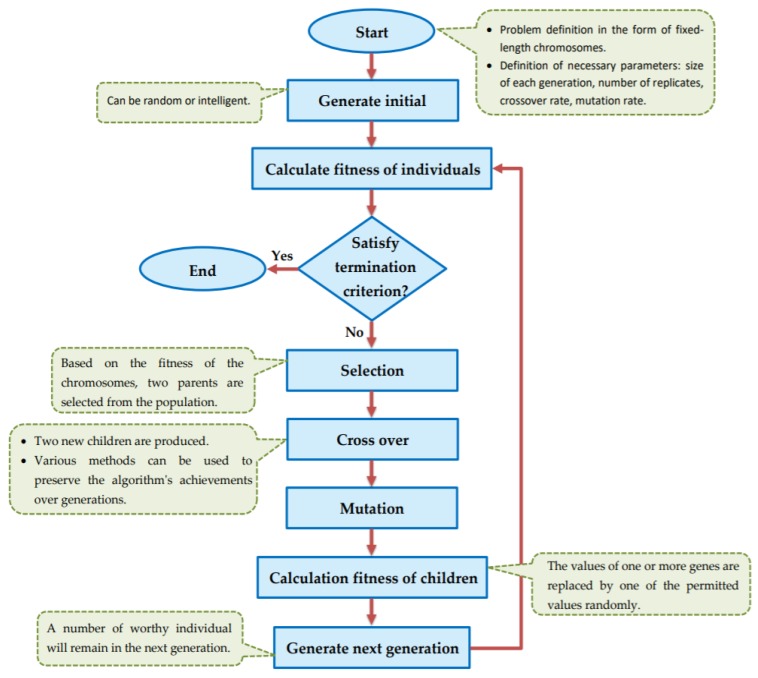
Flowchart of general GA.

Genetic algorithm (GA) is one of the meta-heuristic optimization techniques that use natural evolution selection to find the optimal or a sub-optimal solution. Genetic algorithm uses genotype to show the characteristics of organisms. Genotype space is the space where the chromosomes are encoded and all GA operators are applied to this space. Chromosome evaluation is performed according to the phenotype space, using the fitness function. The general flowchart of the genetic algorithm is shown in Figure 1.

### 2.3. Gradient Boosting Regressor

The gradient boosting regressor (GBR) algorithm is one of the algorithms of the boosting family, which is used for the regression task. This method uses several weak regression decision trees. These trees are trained sequentially, and each subset tree is primarily trained with the data that was mistakenly predicted by the previous tree. This makes the model less likely to focus on simplest samples and focuses more on complex samples. In fact, GBR tries to combine multiple weak learners, which are the weak regression trees, and updates the original learner with gradient descent in each iteration, and ultimately builds a robust model.

### 2.4. Statistical Measurements

We used the criterion of MAD and degree of correlation *R*^2^ between actual age and predicted age to evaluate our age prediction model. The criteria are defined in Table 2. As defined below, m represents number of samples, $y=\left(y^{1},\;y^{2},\;\ldots ,\;y^{m}\right)$ is the actual age values and $\bar{y}$ is the predicted values. All calculations are done with Python 2.7.

### 2.5. Proposed Method

**Figure 2:**
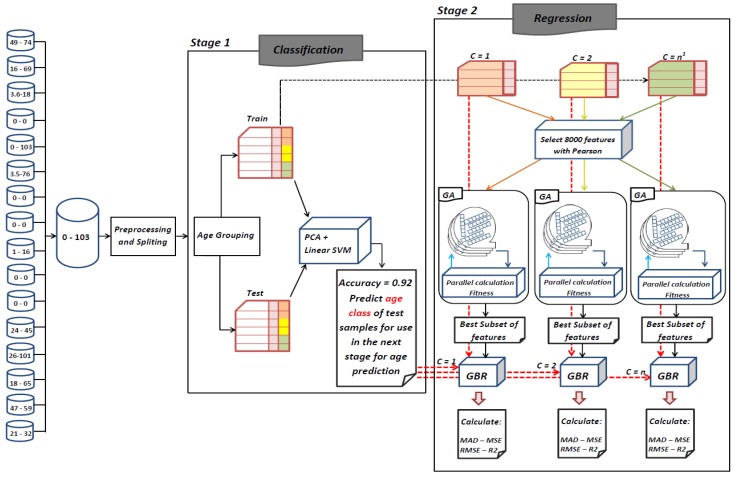
Flowchart of proposed framework. ^1^ *n* denotes number of subgroups for labeling training and test sets.

As depicted in Figure 2, the proposed framework is comprised of two main stages. In the first stage, a classification process is performed. At this stage, samples are first grouped into three age ranges and a new label is added to the data. The classification process is based on this new label. In the second stage, a genetic algorithm is implemented for each age range in order to find the best contributing features for the prediction of the target age range. The rest of this section details these stages.

#### 2.5.1. Classification

Studies have shown that changing patterns of aging varies at different age ranges [36]. Therefore, in the first stage of the proposed method, we look for age ranges that have similar changing patterns. At this stage, a label called the age class is given to the samples of the train and test sets. To find age ranges with the similar changing pattern, we first assume that each decade has similar changes. That is, we considered every sequential 10 years between 0 to 103 as one age class. Thus, we obtained 10 age classes, according to which we labeled the samples as follows.

- Class 1: 0 ≤ ages < 10
- Class 2: 10 ≤ ages < 20
- Class 3: 20 ≤ ages < 30
- Class 4: 30 ≤ ages < 40
- Class 5: 40 ≤ ages < 50
- Class 6: 50 ≤ ages < 60
- Class 7: 60 ≤ ages < 70
- Class 8: 70 ≤ ages < 80
- Class 9: 80 ≤ ages < 90
- Class 10: 90 ≤ ages ≤ 103

**Figure 3:**
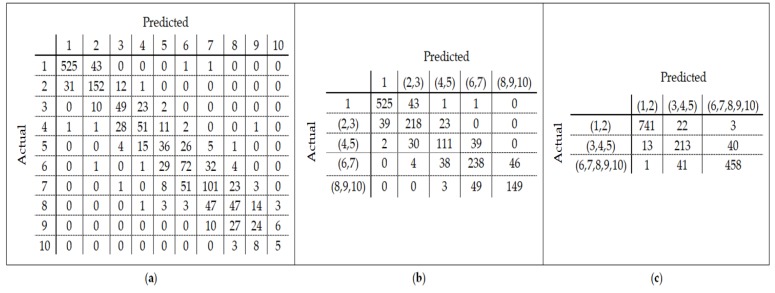
(**a**) Confusion matrix derived from modeling on 10-labeled train data; (**b**) Confusion matrix derived from modeling on 5-labeled train data; (**c**) Confusion matrix derived from modeling on 3-labeled train data.

A linear model using the SVM classifier is then built on these 10 age classes. Since the number of features is high, we reduce the dimensions using PCA before constructing the model. In other words, instead of building a model with 25,000 features, we used 60 new features found by PCA. The accuracy of modeling on 10-labeled data was 0.68 (+/− 0.15). The confusion matrix in Figure 3a shows the age classes that have been misclassified by SVM. By checking out this matrix and observing the similarity between some classes, we performed the data labeling operation again. To this end, classes (1), (2, 3), (4, 5), (6, 7), and (8, 9, 10) were merged respectively. The classification accuracy of SVM for this new class label grouping was 0.80 (+/− 0.23). The confusion matrix of Figure 3b shows the classification errors for the five-class label grouping. Finally, by examining the confusion matrix in the two previous cases, we re-labeled the data according to the three age classes obtained through the integration of classes (1, 2), (3, 4, 5), and (6, 7, 8, 9, 10). The accuracy of the model was 0.92 (+/− 0.12) using these three age groups. The confusion matrix resulting from the modeling on these three age groups is illustrated in Figure 3c. In this paper, three age groups have been considered for relabeling the train and test sets since the changing pattern varies for different age ranges.

In Stage 1, the model is built on the train data that has three classes and 60 features. Afterwards the test data is fed to the model to predict the label of test samples. At the end of this stage, each test sample receives a new label indicating its age range. This label is used in the second stage of the proposed method to predict the age of samples more accurately. Therefore, the higher the classification accuracy at this stage, the lower the error rate in Stage 2.

#### 2.5.2. Regression

In the second stage of the proposed method, the goal is to find CpG-sites that are most relevant to aging in different age ranges. Genetic algorithm has been employed to accomplish this goal. Before selecting features by genetic algorithm, a filter method has used to reduce the search space relatively. To do this, the Pearson correlation coefficient of all the features is calculated and among them the *k* most correlated with age are selected. Finally, using the selected CpG-sites, the exact age of samples in each age range is predicted (three age ranges are found in the previous step). Therefore, contrary to the previous stage, the whole modeling process is performed using the regression task.

The parallel nature of GA enables us to implement the second stage of the proposed framework in a parallel form. The proposed GA for CpG-site selection is parallelized in three parts. Since the data is categorized into three groups based on age class label, the proposed GA is executed for each group separately. Also, evaluation of the chromosomes and calculation of the fitness function is performed in parallel using the MapReduce paradigm.

**Figure 4:**
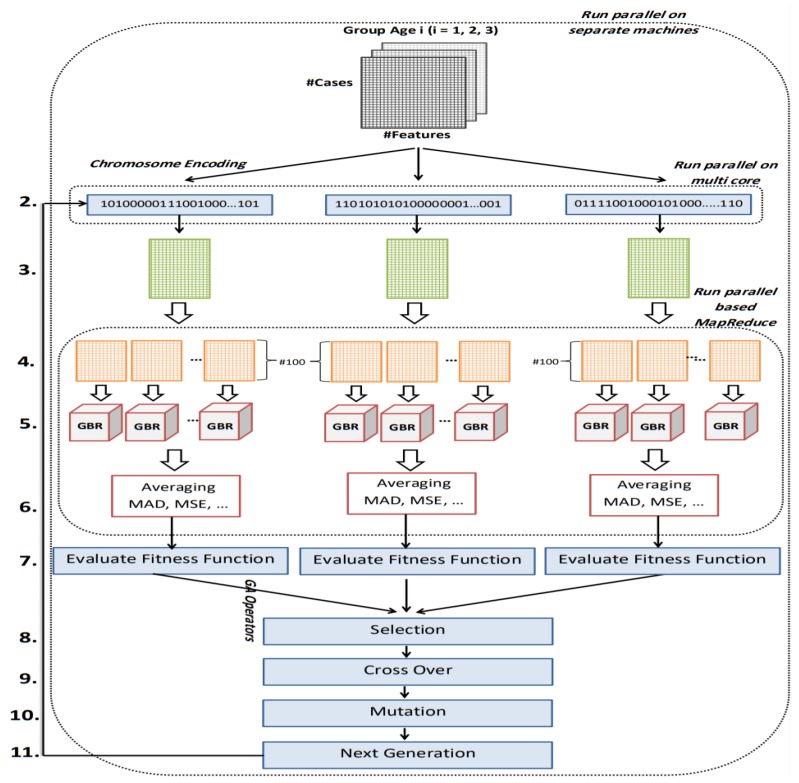
Flowchart of proposed GA.

The first important issue in designing the GA is chromosome encoding. Encoding means representation of the solutions of the problem. We have used binary encoding for this purpose. Each gene in the chromosome has one bit (0 or 1) representing one of the CpG-sites. The length of the chromosomes is the same as the total number of CpG-sites. We filter out from the dataset the CpG-sites whose corresponding genes in the chromosome are zero, and thus the features that remain in the dataset only belong to the CpG-sites whose corresponding genes are equal to one in the chromosome. Next, GBR is employed to evaluate chromosomes based on MAD. The termination criterion we have considered for the genetic algorithm is the number of iterations. Therefore, this process will continue until the condition is satisfied. Figure 4 shows the full details of the proposed parallel genetic algorithm (PGA). The description of each number in Figure 4 is as follows.

**Figure 5:**
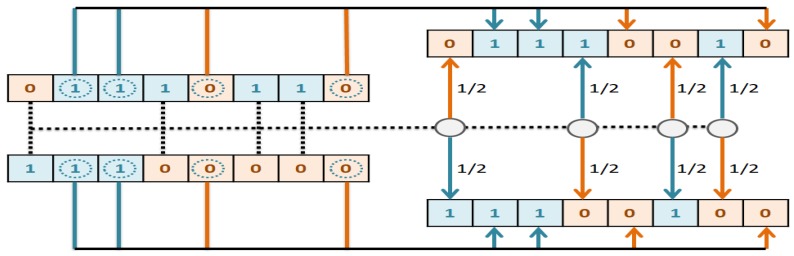
Proposed ParentDifference-based crossover operator.

1. The input of this algorithm is three datasets, each belongs to one age range. These datasets are DNAm matrices. Matrix rows represent samples and columns represent CpG-sites. The first column in each dataset represents the exact age of the samples.
2. Binary method is used to represent chromosomes of genetic algorithm. Value 1 in a gene from the chromosome indicates that the CpG-site corresponding to that gene is selected. Value 0 means deleting CpG-site from the data set. Based on selected CpG-sites by each chromosome, the dataset is filtered and each chromosome generates its own reduced dataset.
3. The reduced dataset contains all samples of the input dataset belongs to age group *i*. Reduction means the filtering of CpG-sites whose corresponding genes in the chromosome have a value 0.
4. The reduced dataset is randomly divided into n parts with 100 samples to perform fitness calculations on these parts in parallel. Figure 5 shows how to do this partitioning.
5. For each partition, one GBR is trained. We have used cross-validation method for evaluating the proposed regression algorithm.
6. At this point, all modeling results (MAD, MSE, RMSE, and R2) are averaged over all partitions of the primary partitioned dataset.
7. We used multi-objective fitness function for each chromosome based two factors: (1) calculated MAD in Step 6; and (2) the number of selected CpG-sites. Since it is a regression problem and the value of MAD has been used in fitness, the lower the value of MAD, the higher the rank of the chromosome. The number of selected CpG-sites are used as a penalty in fitness calculation to make sure that chromosomes that have the fewest selected CpG-sites are ranked higher. The fitness value is calculated from the following formula. According to this formula, for every 50 “1” in the chromosome sequence, one unit is added to the MAD value. That is, fitness value is increased by one unit. As a result, between two chromosomes with the same amount of MAD, the chromosome with the fewest numbers of selected CpG-sites has a better fitness and will have more chance to be selected in next step. The formula for the fitness function is
  (1) $$Fitness=\;MAD+\;\frac{Number\;of\;selected\;CpG-sites\;features}{50}$$
8. After calculating the fitness value of all the chromosomes within the population, the selection operator is applied according to a roulette wheel strategy.
9. The crossover operator applies to the two parents selected in the previous step. We designed a crossover operator that is compatible with our problem. According to this operator, if the value of genes in the same position in the parents was same, the corresponding genes in the offspring would take the same value. However, if the value of genes in the same position in the parent was different, the first child would probably 50% either take value from the first parent or take value from the second parent. Then a complementation value of first child is given to the second child. Figure 5 illustrates the crossover operator logic implemented in this paper.
10. At this point, it is time to perform mutation operator. The standard mutation operator changes the value of each gene of the chromosome with a low probability. However, since the length of the chromosome array is equal to the number of CpG-sites (about 25,000 features), this operation takes much time. In addition, since the number of “0” in a chromosome is much more than “1”, the chances of converting “0” to “1” are greater. With this in mind, we designed the mutation operator as follows: *k* genes from the chromosome are selected for complementation. The way the two genes are selected is that the probabilities of choosing “1” and “0” cells are the same each time.
11. In this step, the next generation chromosomes must be prepared. In the proposed PGA best chromosomes in the current generation are preserved for the next generation. The rest of the next generation chromosomes are determined using the selection and reproduction operators which are described earlier in Steps 8, 9, and 10 of the proposed PGA.

## 3. Results

To evaluate the performance of the proposed MapReduce-based parallel GA, 16 DNAm datasets, all of which are related to human blood tissue, have been tested from two aspects: (1) accuracy test and (2) performance test. Since the data label is age, we have used the GBR regression model. We compared the results of our work with results of recent research works on these 16 datasets. The results of this comparison show that the proposed MapReduce-based parallel GA algorithm outperforms other methods in terms of four regression criteria. On the other hand, we changed the degree of parallelism (number of machines and processing cores) to do the performance test. The results of this test show that using parallelization in the three steps of the algorithm we were able to accelerate the algorithm implementation speed by 71.08 times. All tests are based on the parameters of the genetic algorithm presented in Table 3 and parameters of GBR in Table 4, which are the best combination obtained after several runs based on different parameter combinations.

### 3.1. Accuracy Test

**Figure 6:**
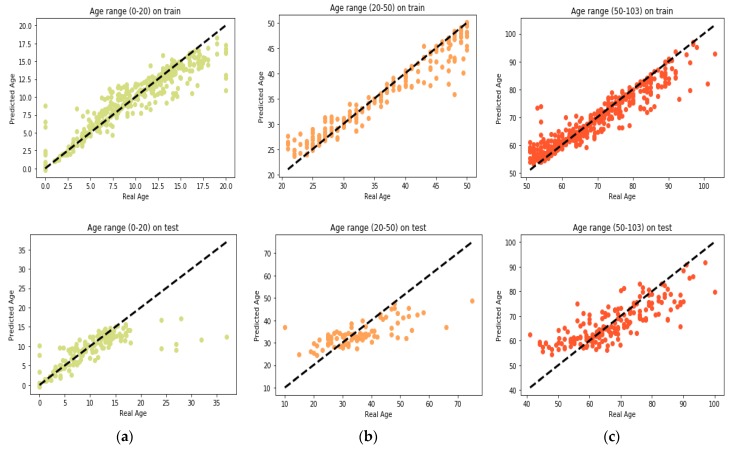
Comparison between the real age and the predicted age by GBR on train and test set (**a**) age range 0–20; (**b**) age range 20–50; (**c**) age range 50–103.

**Figure 7:**
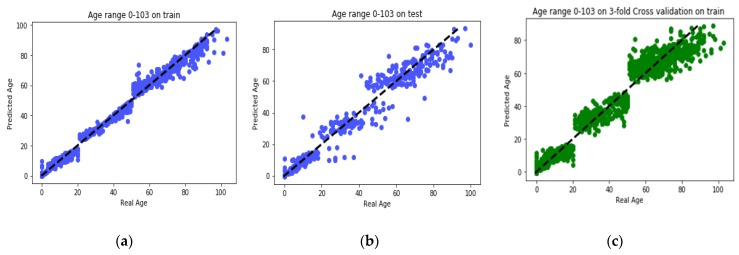
(**a**) Comparison between the real age and the predicted age by GBR on all train set; (**b**) comparison between the real age and the predicted age by GBR on all test set; (**c**) comparison between the real age and the predicted age by 3-fold CV GBR on all train set.

GBR performs its learning process on the features selected by the proposed MR-based PGA. Comparisons between the actual values and the predicted values in each age group are presented in Figure 6. Also, comparison between the actual values and the predicted values on the whole train data by GBR model is presented in Figure 7a. According to this modeling, MAD of the train data was 1.27 years and the correlation between age and DNAm was 99.27%. Also, the MSE and RMSE were 6.33 and 2.51 years, respectively. In Figure 7b, the same comparison is made with all the test data. According to this Figure, the MAD on the test data was 3.62 years and the correlation between age and DNAm was 95.96%. The MSE and RMSE were 35.16 and 5.93 years, respectively. The details of the results obtained by proposed method of this paper and the comparison with GBR model of other study by Li et al. [4] are presented in Table 5.

By comparing the results of GBR model of this paper and the results reported in [4] we found that our proposed method is more accurate. The reason behind this fact is that in each age range we found the features that were most relevant to that age range. The cause of our proposed data label splitting method (the presented three groups for the age class) is that the changing pattern of DNAm is varied in different age ranges. As a result, with age grouping, more reasonable results are obtained. Table 6 presents the details of results for all three age groups. According to this table, we can conclude that DNAm changes in the age range 0–20 years are more regular and these changes become more irregular in the age range of 50–103 years. 

**Figure 8:**
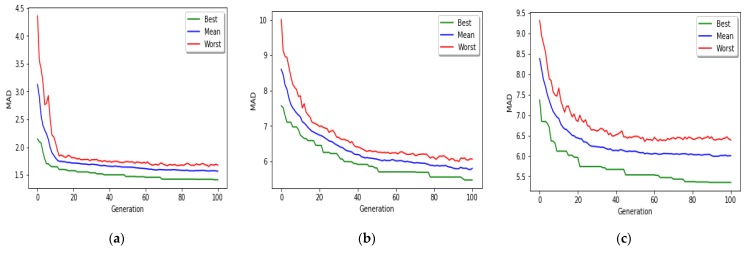
Progress of MR-based PGA, over 100 generations (**a**) age group 0–20 years; (**b**) age group 20–50 years; (**c**) age group 50–103 years.

Figure 8 shows the convergence of the MR-based PGA applied to all three age groups toward the optimal solution. The green lines show the MAD value of the best chromosome in each generation. The red lines represent MAD value of the worst chromosome in each generation. The blue lines show the average MAD of all chromosomes in each generation.

### 3.2. Performance Test

The performance test was done on three machines with Intel Core i7 9xx (Nehalem Class) 2.69 GHz processors and 16 GB RAM memory and 25 GB SSD. To report the results of the performance test, three sets of experiments were done. In the first set, as can be seen in Table 7, the execution time of the proposed method on one and three machines is reported. In this test, each machine has only one core. According to Table 8, the effect of parallelization and the enhancement of parallelism on the chromosomes’ parallel execution were reported. It is noteworthy that in this experiment different age groups were run on different machines. In Table 9, the third set shows the effect of using MapReduce on the fitness calculation of chromosomes. Also, in this experiment different age groups were run on different machines and each machine had 32 processing cores. If all three parts are run concurrently in parallel, the execution time is reduced to 58 min. If none of the parts are run in parallel, the parallelism is one. This means that the algorithm runs on a single machine. In this case, the runtime was about 68 h.

Considering the results presented in the tables above, it can be concluded that with increasing degree of parallelism, the execution time of the algorithm is significantly reduced. Also, the scalability of the proposed algorithm is as much as the scalability of the underlying platform.

### 3.3. Analysis of the Selected CpG-Sites

**Figure 9:**
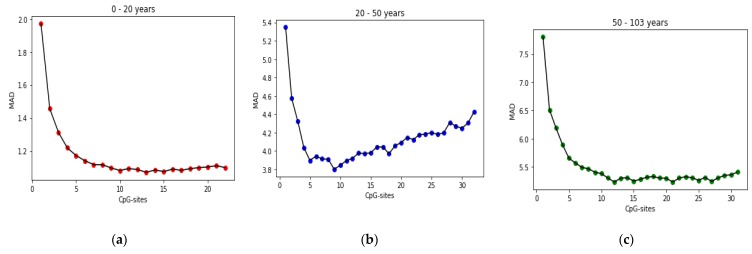
Progress of SFS algorithm during selecting CpG-sites on three age ranges: (**a**) age group 0–20 years; (**b**) age group 20–50 years; (**c**) age group 50–103 years.

The stepwise forward selection (SFS) algorithm is used to evaluate CpG-sites found in each age range, and to rank them. The results reported in the accuracy test section are based on the CpG-sites listed in Table 10 and their order in the table is based on their ranking in the SFS algorithm. In the SFS algorithm, the CpG-sites are added incrementally. Each time the CpG-site is introduced, the selected CpG-sites are tested. When the original introduced CpG-site becomes less significant due to the introduction of the latter CpG-site, it is eliminated. This process is repeated until neither significant CpG-sites are selected into the equation nor significant independent CpG-sites are removed from the regression equation. Figure 9 shows the process of selecting CPG sites by the SFS algorithm.

## 4. Discussion

Most studies in the age prediction field used a simple statistical filter-based feature selection method, such as Pearson correlation for age prediction [1,4,14]. The reason behind selection of the mentioned feature selection method is its simplicity and also rapid operation. However, the problem is that, because they do not use modeling algorithms in their feature selection process, then they usually do not select an acceptable features set and lose many informative features. Another issue that is seen in the feature selection phase of previous studies is that they do not consider the interaction between the selected features set and give each gene a point individually. As a consequence, those approaches are unable to find the set of features that work best together. For these reasons, we used GA to find the best set of CpG-sites that are capable of optimizing MAD of GBR model from real ages. We found that MAD of MR-based PGA achieved superior results in comparison to recent research works.

Bekaert et al. [37] and Alisch et al. [25] found that the relation between DNA methylation and age is not a straight line. Thus, we decided to put together samples where DNAm values change similar to each other, in a group, and build separate models on each group to get better results. Using the SVM classifier and by utilizing the confusion matrix, we divide the dataset to three age groups and consequently, we were able to obtain a better average MAD outcome than that of other previous studies. The results also show that aging pattern is more regular in young ages and very irregular in old ages. As it can be seen in the results of this paper, MAD in the age range of 0–20 years is 1.34 years (on test data) but in the age range of 50–103 is 5.53 years (on test data).

The implemented GA has hundred generations, and in each generation 100 GBR models are evolved for 100 chromosomes. Also, each chromosome has 8000 genes. Therefore, another major issue we faced was the long time it takes to run the feature selection process. The initial runtime of the algorithm took over than 100 h to complete. To solve this problem, we used parallel computing in our algorithm and implemented three parts of the algorithm in a parallel manner with the help of the MapReduce. We also changed some operators of the GA to reduce their execution time. For example, mutation operator in the GA works by generating a random number between [0, 1] for each gene, and the value of a gene changes if the random number was more than the specific threshold. However, running this operator consumes a lot of time for 8000 genes per chromosome. To overcome this issue, we modified the mutation operator to randomly select a fixed number of genes on each chromosome and change their values. Finally, by taking advantage of parallel computing and modifications in some genetic algorithm operators, we lowered the execution time of the algorithm to less than one hour.

Nevertheless, we acknowledge that our approach has some limitations. First, we did not consider the impact of gender on age prediction. Some studies have reported that age-related DNAm may be different in gender [38,39]. Second, we just studied blood tissue methylation patterns since DNAm data on other tissues are limited. Some studies have used DNAm data from teeth and saliva [9,13,37]. If we can use DNAm datasets of different tissues of the body, we can build more complex models using integration methods and get better results on age prediction. Third, our results imply that the ages of samples at the boundaries of groups are poorly predicted. This problem occurred because samples were grouped using crisp age ranges. In future works, one can use fuzzy logic to group samples to get better results for samples at the boundaries of age ranges.

## 5. Conclusions

Predicting human age from genomic data such as DNA methylation is one of the growing areas in recent investigations. An important problem of this data is its high dimensionality, which makes it costly to process. To address this problem, we propose a two-stage parallel algorithm for age prediction. First, the data is clustered into three age groups with similar changing pattern in DNAm. At this stage, each record of the test data is given a new age group label by making use of an SVM classifier. Second, CpG-sites that are correlated with age groups are selected using a rapid parallel genetic algorithm. We take advantage of parallelism in the genetic algorithm to reduce the computational time and cost. To evaluate our method, 16 different datasets in Geo site are used. The MAD of individual age prediction was 1.27 years for the train set and 3.62 years for the test set. Our comparison analysis result suggested better performance of our GBR model than those of the previous works. The performance test also showed acceptable results. According to this test, parallelization of the algorithm was able to reduce the runtime 71.08 times compared to the sequential mode.

## Figures and Tables

**Table 1:** Sixteen healthy blood DNAm datasets.

Availability	DNA Origin	No. Case	Age Range	Citation	Platform
GSE30870	Blood PBMC ^1^	40	(0, 103)	[23]	450 K
GSE32149	Blood PBMC	71	(3.5, 76)	[24]	
GSE36064	Blood PBMC	78	(1, 16)	[25]	
GSE40279	Whole Blood	500	(26, 101)	[26]	
GSE41169	Whole Blood	95	(18, 65)	[27]	
GSE53128	Whole Blood	43	(47, 59)	[28]	
GSE65638	Blood	16	(21, 32)	[1]	
GSE20236	Whole Blood	93	(49, 74)	[29]	27 K
GSE20242	Blood CD4 + CD14	50	(16, 69)	[29]	
GSE27097	Blood PBMC 1	398	(3.6, 18)	[25]	
GSE27317	Blood Cord	168	(0, 0)	[30]	
GSE34257	Blood Cord	84	(0, 0)	[31]	
GSE34869	Blood Cord	24	(0, 0)	[32]	
GSE36642	Blood Cord	123	(0, 0)	[33]	
GSE36812	Blood Cord	48	(0, 0)	[34]	
GSE37008	Blood PBMC	91	(24, 45)	[35]	

^1^ Peripheral blood mononuclear cell.

**Table 2:** Statistical criteria calculated in GBR.

Name	Formula
Mean Absolute Deviation	$MAD=\;\frac{\sum_{i=1}^{m}\left|y{\;}^{i}-\;\bar{y}\right|}{m}$
Mean Square Error	$MSE=\;\frac{\sum_{i=1}^{m}{\left(y{\;}^{i}-\;\bar{y}\right)}^{\;2}}{m}$
Root Mean Square Error	$RMSE=\;\sqrt{\frac{\sum_{i=1}^{m}\left(y{\;}^{i}-\;\bar{y}\right){\;}^{2}}{m}}$
${\mathrm{Correlation}\;\mathrm{Degree}\;R}^{2}$	$R^{2}=1-\;\frac{\sum_{i=1}^{m}{\left(y{\;}^{i}-\;f\left(x^{\;i}\right)\right)}^{\;2}}{\sum_{i=1}^{m}{\left(y{\;}^{i}-\;\bar{y}\right)}^{\;2}}$

**Table 3:** Parameters of proposed MR-based PGA algorithm.

Parameter	Value
Encoding	Binary
String length	8000 selected CpG-sites using Pearson correlation
Generation	100
Population size	100
Selection Method	Roulette wheel
Crossover Method	ParentDifference-based crossover
Mutation method	Presented mutation operator in Step 10 of proposed parallel GA in Section 2.5.2
Elitist strategy	Preserving the top 10 of the best chromosomes in a generation

**Table 4:** Parameters of GBR model.

Parameter	Value
N_estimators	300
Max_depth	4
Min_samples_split	2
Subsample	0.6
Verbose	0
Warm_start	true
alpha	0.6
Learning_rate	0.03
loss	lad

**Table 5:** Comparison of the regression performance between proposed GBR in this paper and proposed GBR by Li et al. [4].

Ref.	Validation Type	MAD	MSE	RMSE	R^2^
		**Train**			
Li et al. [8]	Split	2.7171	20.7243	4.5524	0.9747
MR-based PGA	Split	1.2740	6.3339	2.5167	0.9927
		**Test**			
Li et al. [8]	Split	4.0593	39.8269	6.3109	0.9523
MR-based PGA	Split	3.6233	35.1678	5.9302	0.9596
MR-based PGA	3-fold cross-validation on Train	3.2105	23.9033	4.3927	0.9672

**Table 6:** Comparison between MAD of GBRs made on three age groups.

Age Range	MAD
**3-fold CV on Train**	
0–20	1.4138
20–50	4.1451
50–103	5.3504
**Train**	
0–20	0.6002
20–50	1.3036
50–103	2.2216
**Test**	
0–20	1.3486
20–50	6.1429
50–103	5.5371

**Table 7:** Performance test for the parallel execution of the proposed algorithm for each age group presented in minutes (each age group on one machine). Each machine has only one processing core.

	Using One Machine	Using Three Machines
Minutes	4123	1642

**Table 8:** Performance test for the execution time of the parallel evolution of chromosomes on three machines presented in minutes (each chromosome runs on one processing core).

		Parallelism				
	1	2	4	8	16	32
Minutes	1642	986	608	359	207	126

**Table 9:** Performance test for the execution time of parallel calculation of fitness function using MapReduce on three machines that each machine has 32 processing cores presented in minutes (each mapper runs on one thread).

	Using MR	Without MR
Minutes	**58**	126

**Table 10:** Selected CpG-sites in each three age groups.

Age Range	CpG-Sites
0–20 years	(1) cg14918082, (2) cg27210390, (3) cg01993576, (4) cg19686152, (5) cg19761273, (6) cg13870494, (7) cg19945840, (8) cg09427311, (9) cg17791651, (10) cg06058597, (11) cg10591174, (12) cg23591869, (13) cg21545849, (14) cg15368822, (15) cg20544605, (16) cg03473518, (17) cg09626984, (18) cg03375002, (19) cg00831028, (20) cg08351331, (21) cg16786458, (22) cg19180828.
20–50 years	(1) cg22736354, (2) cg05724065, (3) cg15673110, (4) cg20761322, (5) cg08635242, (6) cg10986043, (7) cg00216361, (8) cg12261786, (9) cg17258195, (10) cg21430666, (11) cg13614181, (12) cg14611174, (13) cg09118625, (14) cg17347389, (15) cg02868123, (16) cg24715735, (17) cg24662961, (18) cg05346899, (19) cg26900154, (20) cg03022541, (21) cg18546419, (22) cg12782180, (23) cg09001953, (24) cg26069252, (25) cg15365950, (26) cg18722841, (27) cg11691938, (28) cg10588377, (29) cg02552572, (30) cg06165395, (31) cg02973263, (32) cg04809787.
50–103 years	(1) cg21296230, (2) cg09809672, (3) cg14094063, (4) cg19560758, (5) cg15297650, (6) cg15399561, (7) cg02228185, (8) cg07944287, (9) cg19945840, (10) cg18815943, (11) cg08005849, (12) cg18113787, (13) cg00635481, (14) cg07091958, (15) cg25809905, (16) cg26508537, (17) cg08395899, (18) cg25671438, (19) cg18630855, (20) cg19722847, (21) cg05361811, (22) cg26526440, (23) cg00915289, (24) cg24490859, (25) cg09462826, (26) cg25490410, (27) cg06885782, (28) cg08158331, (29) cg17022914, (30) cg05140736, (31) cg24110916, (32) cg10940099.
